# Supplementary material for: Removal of a Subset of Non-essential Genes Fully Attenuates a Highly Virulent Mycoplasma Strain
Source: Front Microbiol. 2019 Apr 3;10:664. doi: 10.3389/fmicb.2019.00664 (PMC6456743; doi:10.3389/fmicb.2019.00664)
Supplement: Supplementary file 1 [file Table_1.DOCX]

Supplementary Material

Removal of a subset of non-essential genes fully attenuates a highly virulent *Mycoplasma* strain

Joerg Jores, Li Ma, Paul Ssajjakambwe, Elise Schieck, Anne Liljander, Suchismita Chandran, Michael Stoffel, Valentina Cippa, Yonathan Arfi, Nacyra Asad-Garcia, Laurent Falquet, Pascal Sirand-Pugnet, Alain Blanchard, Carole Lartigue, Horst Posthaus, Fabien Labroussaa, and Sanjay Vashee

*** Correspondence:** Corresponding Author: joerg.jores@vetsuisse.unibe.ch

**Table S1**. **Oligonucleotide primers used to construct the mutagenesis cassettes and to confirm the deletions.**

| **Primer Name** | **Primer sequence (5’🡪 3’)** | **PCR product and notes** | |
| --- | --- | --- | --- |
| D1: Deletion using TREC method (modified CORE3 cassette): | | | |
| FKO-F1 | G**TT GAT TCA GTC TTC ATA ACG TTT TTT ACC TAA AAC ATT TAA TTT TGC ATT** *TAG GGA TAA CAG GGT AA TAC GGA TTA G* | | To amplify the 2.4 kb CORE3 mutagenesis cassette with 50 bp homology overhangs at the target site. |
| FKO-R | **AAT GCA AAA TTA AAT GTT TTA GGT AAA** **AAA CGT TAT GAA GAC TGA ATC AA***T CGG TAC ATA AAT ATA TGT GAT TCT* | |  |
| FKO-F2 | CTG AAG ATT TAG CAG CAG TTA ATC CTC AAA TAC CAA CTA ATA GAA CTA CAG **TTG ATT CAG TCT TCA TAA CGT TT** | |  |
| FKO-DG-F | CCA GTT GTT CCA CCT AAT G | | To amplify 235 bp with RC0421 to confirm the left junction of the glpFKO-CORE3 insertion. To amplify 250 bp with FKO-DG-R to confirm pop-out and seamless deletion of the glpFKO genes. |
| RC0421 | CTT CGG AGG GCT GTC ACC | |  |
| FKO-DG-R | TCG CAT TAG AAA CAA GTA GTC | | To amplify 283 bp with RC0430 to confirm the right junction of the glpFKO-CORE3 insertion. |
| RC0430 | AAG TGT CAC CAT GAA CGA CA | |  |
| D2: Deletion using TREC method (Cre/ URA3 cassette): | | | |
| RC0905 | CTA ATT AAT ATA AAT GAA AGA ATT AAA | | To amplify the 2.4 kb Cre-Ura3 mutagenesis cassette with 50 bp homology overhangs at the target site. |
| RC0906 | TTA AAG AAT TAT TAC TTG GTA TAT TA | |  |
| RC0350 | CGG CTA AAG CTA AAA TCA AAA GAA C | | To amplify 615 bp to confirm the left junction of the gts-CRE/URA3 insertion. To amplify 650 bp with RC0350 and RC0332 to confirm pop-out and seamless deletion of the gts-5kb region. |
| RC0373 | ATA TTT GAG AAG ATG CGG CCA GC | |  |
| RC0332 | AAG AGC ATA TTA GTA AAA TAT TGT CA | |  |
| D3: Deletion using TREC method (CORE3 cassette): | | | |
| lppA123-F1 | **TAA AAC TGG AAA AAA CTA TGA AAA AAG CAA TTA AAT TAT TAC TAT CTA TT***T AGG GAT AAC AGG GTA ATA CGG ATT A* | | To amplify the 2.5 kb CORE3 mutagenesis cassette with 50 bp homology overhangs at the target site. |
| lppA123-R | **AAT AGA TAG TAA TAA TTT AAT TGC TTT TTT CAT AGT TTT TTC CAG TTT TA***T* *CGG TAC ATA AAT ATA TGT GAT TCT* | |  |
| LppA123-F2 | TAG CTA TGT TGT TAT AAT ATT TTC GAA AAA TTT TTT GTA GGT ATA TTA TT**T AAA ACT GGA AAA AAC TAT GAA AAA AG** | |  |
| lppA123-DG-F | CTA GAT GTA AAT TAC CAC TAG G | | To amplify 360 bp with RC0421 to confirm the left junction of the lppA-CORE3 insertion. To amplify 436 bp with lppA-DG-R to confirm pop-out and seamless deletion of the lppA region. |
| lppA123-DG-R | GGT TTT GAT GGT GTT TTT GGA G | | To amplify 237 bp with RC0430 to confirm the left junction of the lppA-CORE3 insertion. |
| D4: Deletion using TREC method (CORE3 cassette): | | | |
| 50CORE-F1 | **TTTTAATCCTCCAACCTATTAATATTTTAAATAAGATAAAACATTGTTGG***TAGGGATAACAGGGTAATACGGATTAG* | | To amplify the 2.5 kb CORE3 mutagenesis cassette with 50 bp homology overhangs at the target site. |
| 50CORE-R | CCAACAATGTTTTATCTTATTTAAAATATTAATAGGTTGGAGGATTAAAA*TCGGTACATAAATATATGTGATTCTG* | |  |
| 50CORE-F2 | AGTATAAAACTTTTATCCTAACCGATTTTAAGTTTTATACAGGAGGAATT**TTTTAATCCTCCAACCTATTAATATTT** | |  |
| RC0863 | TCAACATATTCTGGTATGTCT | | To amplify 425 bp with RC0421 to confirm the left junction of the ICE-CORE3 insertion. To amplify 480 bp with RC0878 to confirm pop-out and seamless deletion of the ICE region. |
| RC0878 | TAATCTAACACAACCGGTAG | | To amplify 325 bp with RC0430 to confirm the left junction of the ICE-CORE3 insertion. |
| D5: Deletion using TREC method (CORE3 cassette): | | | |
| TREC37-F1 | **TAATTCATTAGTCTCTAATTCTTATTAAAAGATTTAGAGATTTTTTATTT***TAGGGATAACAGGGTAATACGGATTAG* | | To amplify the 2.5 kb CORE3 mutagenesis cassette with 50 bp homology overhangs at the target site. |
| TREC37-R | **AAATAAAAAATCTCTAAATCTTTTAATAAGAATTAGAGACTAATGAATTA***TCGGTACATAAATATATGTGATTCT* | |  |
| TREC37-F2 | CTAGAAATATATTAATATATCATTAAAAATAAAAATAGCATTTAAGATT**TAATTCATTAGTCTCTAATTCTTATTAA** | |  |
| TREC37-DG-F | TGGTGTTGCTACTGAAATATG | | To amplify 250 bp to confirm pop-out and seamless deletion of the D5 region. |
| TREC37-DG-R | ACTTAAAAAAGAACGACACCG | |  |
