# Supplementary material for: Removal of a Subset of Non-essential Genes Fully Attenuates a Highly Virulent Mycoplasma Strain
Source: Front Microbiol. 2019 Apr 3;10:664. doi: 10.3389/fmicb.2019.00664 (PMC6456743; doi:10.3389/fmicb.2019.00664)
Supplement: Supplementary file 2 [file Table_2.DOCX]

Supplementary Material

Removal of a subset of non-essential genes fully attenuates a highly virulent *Mycoplasma* strain

Joerg Jores, Li Ma, Paul Ssajjakambwe, Elise Schieck, Anne Liljander, Suchismita Chandran, Michael Stoffel, Valentina Cippa, Yonathan Arfi, Nacyra Asad-Garcia, Laurent Falquet, Pascal Sirand-Pugnet, Alain Blanchard, Carole Lartigue, Horst Posthaus, Fabien Labroussaa, and Sanjay Vashee

*** Correspondence:** Corresponding Author: joerg.jores@vetsuisse.unibe.ch

**Table S2**. **Body temperature of goats in Celsius measured throughout the trial.**

| **Infected with** | **GM12::YCpMmyc1.1-*D68*** | | | | | |
| --- | --- | --- | --- | --- | --- | --- |
| **Animal ID** | **CK035** | **CK045** | **CK047** | **CK049** | **CL001** | **CL003** |
| 0 dpi | 38.2 | 38.4 | 37.6 | 37.7 | 37.7 | 38.6 |
| 1 dpi | 38.2 | 38.8 | 37.9 | 38.4 | 37.9 | 38.5 |
| 2 dpi | 38.5 | 38.5 | 38.4 | 38.4 | 39.1 | 38.1 |
| 3 dpi | 38.2 | 38.2 | 38.4 | 37.9 | 38.3 | 38.2 |
| 4 dpi | 38.3 | 38.6 | 38.6 | 38.6 | 37.8 | 38.7 |
| 5 dpi | 38.3 | 38.6 | 38.2 | 38.9 | 38.3 | 38.6 |
| 6 dpi | 39.0 | 39.1 | 39.1 | 38.5 | 38.7 | 39.4 |
| 7 dpi | 38.1 | 38.8 | 38.4 | 38.5 | 37.9 | 38.5 |
| 8 dpi | 39.0 | 38.9 | 38.5 | 38.6 | 38.3 | 38.6 |
| 9 dpi | 38.8 | 39.4 | 38.5 | 38.1 | 37.9 | 37.9 |
| 10 dpi | 38.5 | 38.5 | 38.0 | 38.0 | 37.7 | 38.0 |
| 11 dpi | 38.5 | 39.5 | 37.3 | 38.3 | 37.8 | 38.2 |
| 12 dpi | 38.3 | 38.9 | 38.7 | 39.2 | 38.1 | 38.3 |
| 13 dpi | 39.1 | 38.7 | 38.8 | 38.8 | 38.1 | 38.8 |
| 14 dpi | 39.5 | 38.9 | 38.7 | 38.7 | 37.8 | 39.0 |
| 15 dpi | 38.2 | 38.2 | 38.2 | 37.9 | 38.4 | 38.2 |
| 16 dpi | 38.9 | 38.4 | 38.2 | 38.5 | 37.8 | 38.4 |
| 17 dpi | 38.4 | 38.5 | 38.1 | 38.6 | 38.2 | 38.3 |
| 18 dpi | 38.6 | 38.0 | 38.2 | 38.2 | 38.0 | 38.3 |
| 19 dpi | 38.8 | 38.8 | 38.9 | 38.7 | 38.3 | 38.8 |
| 20 dpi | 38.7 | 38.4 | 38.4 | 38.2 | 37.9 | 38.2 |
| 21 dpi | 38.9 | 38.7 | 38.8 | 38.8 | 37.9 | 38.4 |
| 22 dpi | 38.5 | 38.4 | 37.9 | 38.1 | 37.4 | 38.4 |
| 23 dpi | 38.9 | 38.6 | 38.8 | 38.4 | 38.2 | 38.3 |
| 24 dpi | 38.5 | 38.4 | 37.7 | 38.1 | 37.8 | 38.4 |
| 25 dpi | 38.2 | 38.1 | 38.2 | 38.5 | 37.9 | 38.6 |
| 26 dpi | 38.6 | 38.5 | 38.2 | 38.2 | 38.2 | 38.7 |
| 27 dpi | 38.4 | 38.3 | 38.1 | 38.6 | 38.4 | 38.7 |
| 28 dpi | 38.2 | 38.6 | 38.6 | 38.3 | 37.9 | 38.4 |

| **Infected with** | **GM12** | | | | | | | |
| --- | --- | --- | --- | --- | --- | --- | --- | --- |
| **Animal ID** | **CK032** | **CK034** | **CK040** | **CK043** | **CK046** | **CK048** | **CK051** | **CL002** |
| 0 dpi | 38.0 | 38.3 | 38.5 | 38.1 | 38.6 | 38.2 | 38.8 | 38.4 |
| 1 dpi | 38.6 | 38.5 | 38.5 | 38.0 | 38.8 | 38.2 | 38.6 | 37.9 |
| 2 dpi | 40.0 | 39.3 | 39.7 | 38.2 | 39.8 | 40.6 | 39.0 | 39.3 |
| 3 dpi | 40.4 | 40.0 | 40.2 | 39.8 | 41.2 | 41.0 | 40.3 | 39.8 |
| 4 dpi | 40.7 | 41.3 | 40.9 | 40.5 | 41.4 | 41.5 | 41.3 | 40.7 |
| 5 dpi | 40.2 | 42.0 | 40.0 | 40.5 | 41.5 | 41.5 | 41.1 | 40.8 |
| 6 dpi | 40.4 |  |  |  |  |  |  |  |

dpi-days post infection
