# Supplementary material for: Removal of a Subset of Non-essential Genes Fully Attenuates a Highly Virulent Mycoplasma Strain
Source: Front Microbiol. 2019 Apr 3;10:664. doi: 10.3389/fmicb.2019.00664 (PMC6456743; doi:10.3389/fmicb.2019.00664)
Supplement: Supplementary file 3 [file Data_Sheet_1.PDF]

*Supplementary Material*

# Removal of a subset of non-essential genes fully attenuates a highly virulent *Mycoplasma* strain

**Joerg Jores, Li Ma, Paul Ssajjakambwe, Elise Schieck, Anne Liljander, Suchismita Chandran, Michael Stoffel, Valentina Cippa, Yonathan Arfi, Nacyra Asad-Garcia, Laurent Falquet, Pascal Sirand-Pugnet, Alain Blanchard, Carole Lartigue, Horst Posthaus, Fabien Labroussaa, and Sanjay Vashee**

*** Correspondence:** Corresponding Author: joerg.jores@vetsuisse.unibe.ch

**Figure S1**.

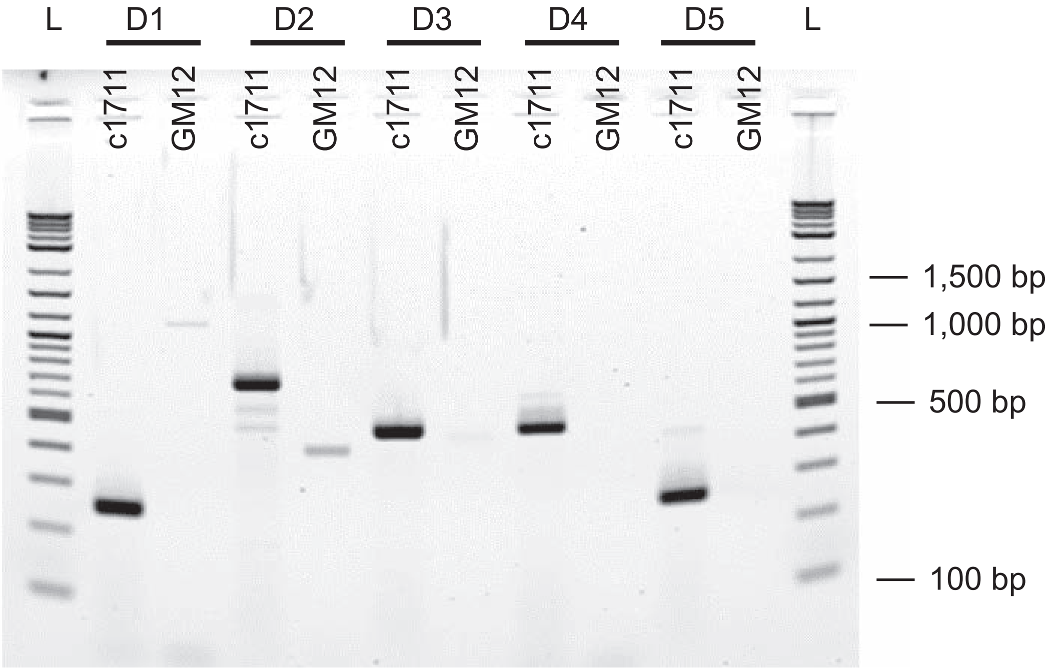


**Figure S1:** Specific PCR confirming the deletions in GM12::YCpMmyc1.1-*Δ68*. Genomic DNA from GM12::YCpMmyc1.1-*Δ68* (clone 1711) and its parental strain wtGM12 was used as template. Specific primers were used to amplify across the deleted regions. DNA from GM12::YCpMmyc1.1-*Δ68* (clone 1711) yielded expected amplicons that are a product covering the flanking regions of the deleted subgenomic fragment. Diagnostic primers FKO-DG-F and FKO-DG-R result in a 250 bp amplicon across the D1 region in clone 1711, primers RC0350 and RC0332 result in a 650 bp amplicon across the D2 region, primers LppA123-DG-F and LppA123-DG-R result in a 436 bp amplicon across the D3 region, primers RC0863 and RC0878 result in a 480 bp amplicon across the D4 region, and primers TREC37-DG-F and TREC37-DG-R result in a 250 bp region across the D5 region. L denotes the DNA ladder.
